# Growth and mechanical correlations of calcified cartilage in Batoidea: A histomorphological study using the *Raja asterias* model

**DOI:** 10.1111/jfb.16037

**Published:** 2025-01-02

**Authors:** Ugo E. Pazzaglia, Marcella Reguzzoni, Genciana Terova, Fabrizio Serena, Cecilia Mancusi, Guido Zarattini, Piero A. Zecca

**Affiliations:** ^1^ Department of Medical and Surgical Specialties, Radiological Sciences and Public Health University of Brescia Brescia Italy; ^2^ Department of Medicine and Technological Innovation University of Insubria Varese Italy; ^3^ Department of Biotechnology and Life Sciences University of Insubria Varese Italy; ^4^ Institute of Marine Biological Resources and Biotechnology National Research Council (CNR‐IRBIN) Mazara del Vallo Italy; ^5^ Marine Resources Environmental Protection Agency of Tuscany Region (ARPAT) Livorno Italy

**Keywords:** Batoidea skeletal growth, “crustal,” “catenated,” “3D network”, locomotion type, mechanical demand, mineralization patterns

## Abstract

This study investigates the growth and calcification of the appendicular skeleton in *Raja asterias* (Delaroche, 1809), a member of the Batoidea, to explore the relationship between histomorphology and the mechanics of batoid locomotion within the water column. Although much prior research has focused on the “tessellated pattern” in these fishes, the variable structure of the appendicular skeleton provides fresh insights into the understudied interplay between skeletal histomorphology and the mechanical functions of Batoidea fins. The shape and initial growth of fin cartilage are influenced by the orientation of chondrocyte mitoses prior to mineral deposition, with subsequent calcification playing a pivotal role in shaping skeletal architecture. This study documents two distinct growth patterns: “crustal” and “catenated.” The crustal pattern is predominantly observed in larger skeletal elements, such as the central body structures (skull, rostrum, and jaws), girdles, pterygia, and compound radials, whereas fin radials follow the catenated growth pattern. Notably, early‐stage chondrichthyan cartilage shares similarities with mammalian metaphyseal growth plate cartilage, though in chondrichthyans, the calcified matrix is not resorbed or replaced by bone. Additionally, a previously unrecognized calcification pattern is identified in the pelvic‐fin radials of *R. asterias*, indicating that the mechanical demands of locomotion in the water column may have driven the evolution of variable fin flexibility in Batoidea. This flexibility is achieved through joint mobility (diarthroses and amphiarthroses), specialized fin structures, and the distinct calcification patterns of the pectoral and pelvic fins.

## 1 INTRODUCTION

The morpho‐mechanical basis of vertebrate locomotion, whether on land, in water, or in the air, relies on the development of rigid skeletal segments interconnected by flexible joints. Skeletal stiffness is achieved through the incorporation of minerals into the organic matrix of cartilage or bone in both mammals and fish, although a distinction exists between the subclasses Osteichthyes and Chondrichthyes in fish.

During embryonic development, cartilage is the first tissue to differentiate from the mesenchyme forming the anlage—the early scaffold on which each skeletal segment is built—(Hall, 2005). In mammals, bone matrix appears later during foetal development following two distinct patterns of ossification. The first, known as primary ossification, occurs through the differentiation of osteoblasts within a connective tissue matrix. The second, termed endochondral ossification, involves the deposition of bone matrix onto a preexisting scaffold of calcified cartilage. This latter process is the most common pattern in vertebrates and leads to the formation of multiple ossification centers within the cartilage model (Pazzaglia et al., 2014, 2017, 2018).

The noncalcified cartilage strips that persist between the diaphyseal and epiphyseal ossification centers, known as metaphyseal growth plate cartilage, allow for longitudinal growth of skeletal segments. This process continues until chondrogenesis slows and ceases after puberty, as extensively documented in *Homo sapiens* and tetrapods.

The histology of the metaphyseal growth plate is characterized by three key processes: (1) the orderly alignment of vertically oriented chondrocyte mitoses; (2) the formation of intercolumnar septa in which mineral deposition subsequently occurs; and (3) the invasion of metaphyseal vessels from the diaphyseal vasculature, followed by the differentiation of osteoblasts, which begin depositing bone matrix on the calcified cartilage septa. This highly organized cellular arrangement forms the biological foundation for skeletal growth in mammals and birds.

In Osteichthyes, skeletal segment growth is driven by primary matrix deposition by osteoblasts and subsequent remodeling (Mackie et al., 2008; Witten & Huysseune, 2009), though this process lacks the localized structure seen in mammalian metaphyseal growth plates. In contrast, Chondrichthyes exhibit a distinct pattern of skeletal segment stiffening and growth, where calcified cartilage is not replaced by bone and does not undergo remodeling (Cerny et al., 2004; Gillis et al., 2009). This provides an alternative model to endochondral ossification, challenging traditional views of vertebrate skeletal growth.

Mechanobiological research has largely focused on the adaptation of the vertebrate skeleton to growth and mechanical stress through endochondral ossification and remodeling processes (Frost, 2003; Huiskes, 2000; Nauen & Lauder, 2002; Pivonka, 2017; Wolff, 1892). The goal of this study is to document the alternative model of growth and skeletal stiffening in *Raja asterias* (Delaroche, 1809), a member of the Chondrichthyes subdivision Batoidea, which has evolved unique mechanisms for skeletal development.

## 2 MATERIALS AND METHODS

### 2.1 Ethics statement

No approval from the Institutional Ethics Committee was required for this study, as the specimens, 2 to 6 months old at the time, were found deceased in the fish pond overnight. They were collected the following morning and preserved in 10% buffered formaldehyde within 12 h. Handling of these samples did not necessitate ethical authorization.

### 2.2 Study population

The study focused on both juvenile and adult specimens of the oviparous *R. asterias*, a species classified as near threatened in the Mediterranean (Abella & Serena, 2005; Dulvy et al., 2016; Serena et al., 2010; Serena et al., 2020). Six juvenile specimens (Table 1, numbers 1–6) were obtained from the Livorno Aquarium in Italy. These juveniles were born from internally fertilized eggs, which were encased in flat, rectangular, leathery capsules with tendrils at the corners, aiding in anchorage. After hatching from egg cases laid on a sandy substrate in September 2022, the juveniles were reared in seawater maintained at a constant temperature of 18°C. The size at sexual maturity for this species is 56.1–66 cm total length (TL) for females and 45–54 cm TL for males, typically reached at 3–4 years of age. Embryonic development takes approximately 5–6 months, with the size at birth being 8–9 cm TL. The juveniles examined in this study ranged in age from approximately 2 months (specimen number 1), 3–5 months (specimen numbers 2–4), to 6 months (specimen numbers 5–6). Specimens that died overnight in the fish pond were collected the following morning and then fixed in a 10% buffered formaldehyde solution within 12 h. No authorization from the Institutional Ethics Committee is required for the processing of these samples.

**TABLE 1:** Data of the studied *Raja asterias* population, divided into age groups based on weight, disk width, and length.

No.	Age group	Approximate age (months)	Sex	Weight (g)	Disk width (cm)^a^	Length (cm)	Number of pectoral‐fin rays^b^
1	A	2	n.d.	4.2	5.5	8.2	n.d.
2	B	3–5	n.d.	12.3	8.7	11.5	n.d.
3	B	3–5	n.d.	14.1	8.5	13	n.d.
4	B	3–5	n.d.	20.0	11.5	15	n.d.
5	C	6	n.d.	50.9	13	16.5	n.d.
6	C	6	n.d.	90.5	16	20	n.d.
7	D	Over 6	M	123	19	29	70
8	D	Over 6	F	179	21.5	30.5	69
9	D	Over 6	M	343	27	40	70
10	D	Over 6	M	769	34	52.5	71

^a^ The width of the pectoral fin was measured from the tip of the left pectoral fin to that of the corresponding right fin.

^b^ The number of pectoral‐fin rays was determined in age group D using X‐ray images; in groups A–C, the number of rays could not be determined (n.d.) with transmitted light.

The four adult specimens (Table 1, numbers 7–10) were captured during a scientific campaign conducted by the ARPAT team (Livorno) in the Tyrrhenian Sea, a part of the northwestern Mediterranean. This research was carried out as part of the Fisheries Data Collection Programme (DCF), funded by the Italian Ministry of Agriculture, Food and Forestry (MIPAAF, now MASAF—Ministry of Agriculture, Food Sovereignty and Forestry) and the EC.

### 2.3 Preparation and selection of anatomical specimens

Radiographs (dorso‐ventral projection) of the entire fish were taken prior to sample preparation. The rostrum, head, and vertebral column were separated from the appendicular skeleton using two parallel longitudinal cuts isolating the pectoral and pelvic fins, as well as the tail. The pectoral and pelvic girdles were bisected along the midline. In adult specimens, the dorsal skin of the right pectoral and pelvic fins was removed to obtain higher‐resolution radiographs in dorso‐ventral projection. To further investigate the architecture of the calcified skeletal segments, a deeper dissection was performed in which subcutaneous tissue and muscles were removed. This was documented by low‐power microscopy of the unstained specimens (immersed in buffered phosphate solution, in large Petri dishes photographed under transmitted light). A peripheral skin band of about 2 cm was left intact to prevent damage during manipulation of the delicate apical radials. The right fins were sectioned in their entire thickness and prepared for light microscopic histology. A full‐thickness strip of the pectoral fin, including both dorsal and ventral skin, was cut along the mid‐lateral axis from the specimen with the largest disk width (e.g., specimen D, *n* = 10) to document the thickness of the muscle layer from the pterygia to the fin tip. The pelvic fins were processed similarly.

### 2.4 Transillumination, histology, and heath deproteination

**FIGURE 1:**
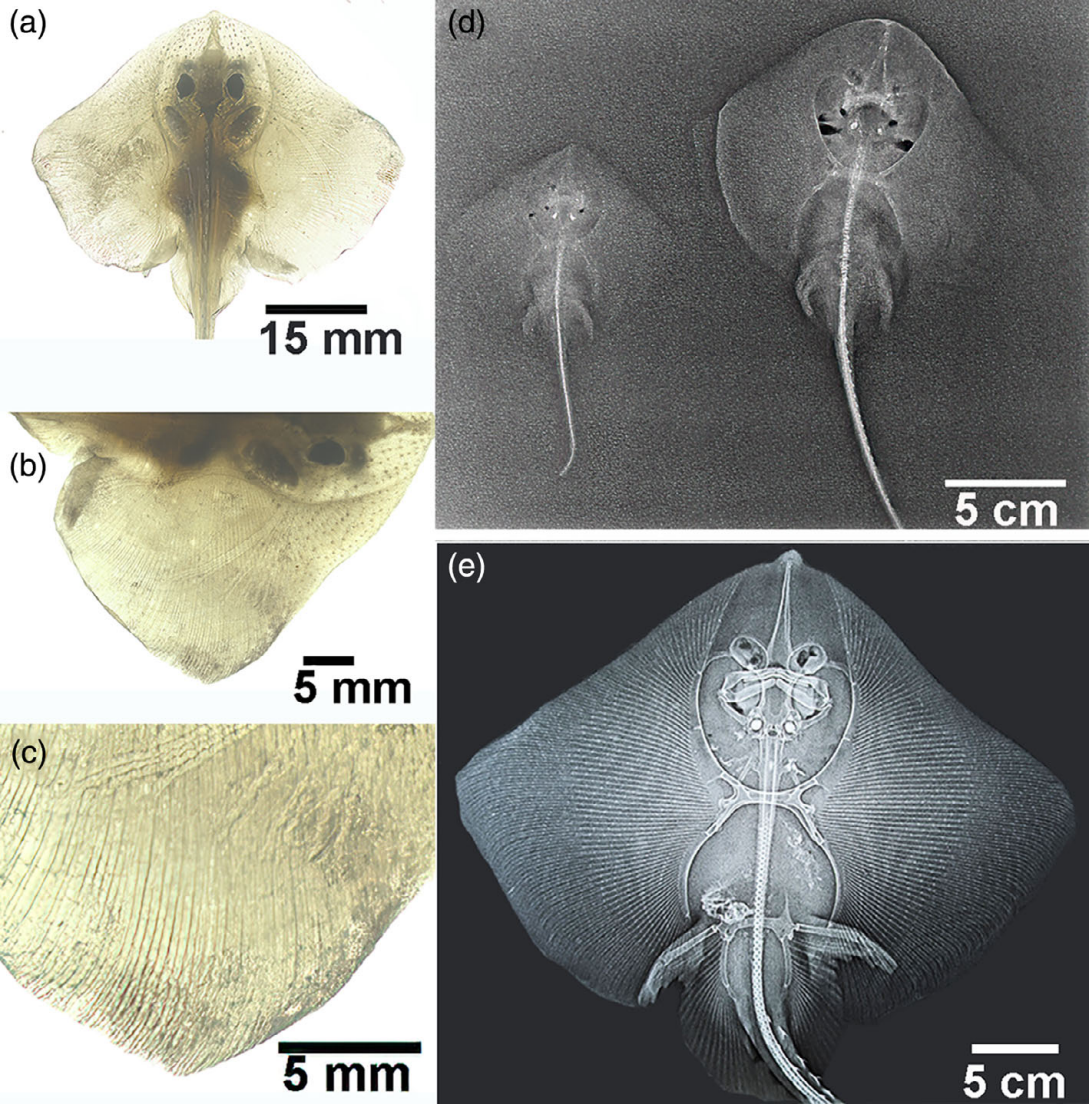
Transillumination and X‐ray images of *Raja asterias* across different age groups. (a–c) *R. asterias* (specimen 1, age group A). (a) The fish body under transillumination in a dorso‐ventral projection. (b, c) The right pectoral fin at a higher magnification. In the thinner lateral sector (c), a line pattern is visible, though individual radials are indistinct due to the minimal mineral deposition. (d) *R. asterias* (specimen 3, age group B and specimen 5, age group C). X‐rays in a dorso‐ventral projection reveal an increase in size and mineral deposition in the appendicular skeleton segment, as well as in the spine, mandible, and rostrum. (e) *R. asterias* (specimen 9, age group D). X‐rays in a dorso‐ventral projection demonstrate advanced mineralization in all skeletal elements with clearly distinguishable radial patterns in the medial and lateral sectors of the pectoral fins.

The left fins of the first three specimens (numbers 1–3) were photographed under transmitted light without any skin preparation. In contrast, the left fins of the older specimens (numbers 4–10) were viewed using the same technique after the removal of the skin and muscle layers (Figure 1a–c).

The selected left fins for histological analysis were decalcified for 30 days in a 2% acetic‐hydrochloric acid solution, then dehydrated through a series of increasingly concentrated ethanol‐water solutions, and finally embedded in paraffin. Transverse and longitudinal sections, 7 μm thick, were cut using a microtome and stained with haematoxylin–eosin, Masson trichrome, May‐Grunwald‐Giemsa, periodic acid–Schiff (PAS), and Alcian blue. Non‐decalcified specimens were embedded in Technovit 7200 resin (Kulzer GmbH, Germany), and 150‐μm‐thick sections were prepared using the Exact cutting/grinding system (Exact Advanced Technology GmbH), then stained with von Kossa or methylene blue acid fuchsin. These sections were examined using an Olympus SZX7 stereomicroscope and an Olympus BX51 microscope (Olympus Ltd., Tokyo, Japan). Additionally, samples deproteinized at 400 and 1200°C were mounted on glass slides for microscopic observation without staining.

### 2.5 Morphometry

The growth patterns of the *R. asterias* population studied were determined based on body weight, width of the pectoral disk, and TL from the rostrum to the caudal fin, and the individuals were divided into four arbitrary age classes (Table 1). The number of rays in the pectoral and pelvic fins could only be accurately counted in the dorso‐ventral radiographs of the left lateral fins of the oldest specimens (age group D, number 10). The number of radials/ray and the number of tiles/radial could only be accurately counted in the right pectoral fin using transillumination and low‐power microscopy after the muscles were manually dissected and cleaned in hydrogen peroxide (H_2_O_2_) 50%, which is an extremely powerful oxidizer. These parameters were not applicable to the pelvic fins of the same specimen due to the different calcification patterns of the radialis.

Morphometric analysis was performed on the right pectoral fin of specimen 10 (Table 1) using transillumination and low‐power microscopy. Measurements focused on the five central rays of the lateral sector and their sequence of radials from the most apical to the next nine toward fish's midline, as these correspond to true mono‐columnar radials (Pazzaglia, Reguzzoni, Milanese, et al., 2023). Two parameters were assessed: (1) the length of the radials (i.e., the distance between the medial and lateral plates at the radial ends of each column) and (2) the number of calcified tiles per radial.

For specimens in age groups A, B, and C, the radial length and number of tiles could not be quantified due to the low density and compactness of the mineral deposits. However, these specimens provided a qualitative description of the early calcification process. Despite these limitations, the structure of the apical ray tip, characterized by a layer of flexible keratin rods located between the dorsal and ventral skin, was documented using transillumination in all specimens from age groups A, B, and C. In age group D, the length of the central column could be measured across all radials due to the presence of terminal plates (the first to calcify), but only in specimen 10 were the aligned plates sufficiently marked to be counted.

The rays of the pelvic fin exhibited a different segmentation and calcification pattern compared to the pectoral fins, making direct comparative morphometric analysis between these two skeletal elements unfeasible. The length measurements of the radials were carried out with the programme Cells (Soft Imaging System, GmbH, Germany). The variation in the number of columns of the right pectoral fin was plotted with the corresponding length of the single‐column radials in order from medial to lateral of the right pectoral fin of specimen 10 (age group D), selecting the five central rays of the lateral sector. The graph was created using the Excel programme (Microsoft, Redmont, Washington, USA), to illustrate the variation in the two parameters from the outer edge to the midline of the vertebral column.

## 3 RESULTS

### 3.1 Mechanical model

The mechanical model of the Rajidae skeleton can be simplified to a central, stiff, and bulky structure composed of the following components from front to back: rostrum, neurocranium, vertebral column (including the cervical and thoracic synarcual), and pectoral and pelvic girdles. The pectoral girdle is connected to the rigid pterygial axis by very mobile diarthroses and further to the fin ray system (characterized by less mobile, interradial amphiarthroses). This remarkable anatomical design, consistent with the mechanical requirements, provides the essential system for the flapping movement of the “wings” (Figures 1e and S1). The posterior vertebral column, along with the dorsal and caudal fins, has limited mobility, which is facilitated by the intervertebral joints (Compagno, 1999).

In *R. asterias*, the primary propulsive force for movement within the water column is generated by the flapping and undulating motion of the symmetrical right and left pectoral fins, with complementary support from the pelvic fins. Unlike other elasmobranchs, such as sharks, or Batoidea *Torpedo marmorata*, the posterior vertebral segment and tail contribute only minimal axial thrust (Pazzaglia, Reguzzoni, Manconi, et al., 2023).

Furthermore, this study documents a significant difference in mechanical performance between the pectoral and pelvic fins of *R. asterias*. This difference is evidenced not only by the much larger surface area of the pectoral fins (approximately 25 times larger than the pelvic fin) but also by variations in the number and orientation of rays, the shape of the radials, and the size, structural morphology, and mass of the dorsal and ventral muscles.

### 3.2 Morphometry

#### 3.2.1 Pectoral fin

**FIGURE 2:**
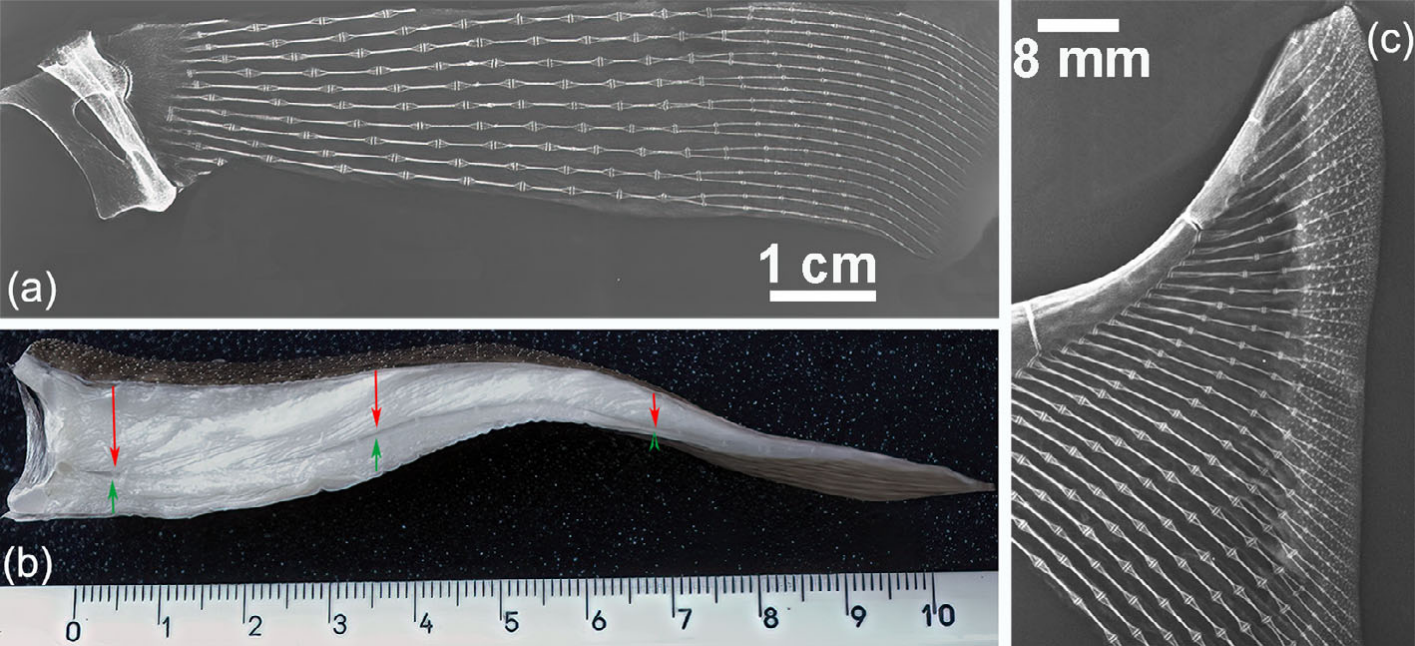
X‐rays of the central and anterior sectors of the pectoral‐fin rays (*Raja asterias*, specimen 10, age group D) and a longitudinal full‐thickness section of the fin. (a) Transformation at approximately mid‐ray length in the medial sector from a vertical, (bi‐columnar) radial pattern to independent, horizontal (mono‐columnar) radials laterally. (b) Full‐thickness section of a central ray showing muscle layers (red arrows) and ventral layers (green arrows), with thickness decreasing from medial to lateral. The dorsal layer is thicker than the ventral, and muscles are not evident in the fins' marginal band. (c) Reduction in the number of radials in the anterior and posterior sector rays of the fin.

In the oldest specimen studied (number 10, age group D), 70 rays were counted in the left pectoral fin. The length of the rays gradually decreased from the five central rays to the anterior and posterior fin sectors (Figures 1e and 2a,c). Two key parameters were measured in the central sector: (1) radial length (the distance between the medial and lateral plates at the ends of each radial, which corresponded to the basal layer of the amphiarthroses joint) and (2) the number of calcified plates in the radial. Radial length increased steadily along each ray from lateral to medial in both the medial and lateral sectors. However, the number of calcified tiles/radial could only be counted in the lateral sector, as the mono‐columnar arrangement was an essential prerequisite for a reliable count. The number of calcified tiles/radial showed an irregular variation that did not correlate with the regular radial length reduction from lateral to medial (Figures S2 and S3).

#### 3.2.2 Pelvic fin

**FIGURE 3:**
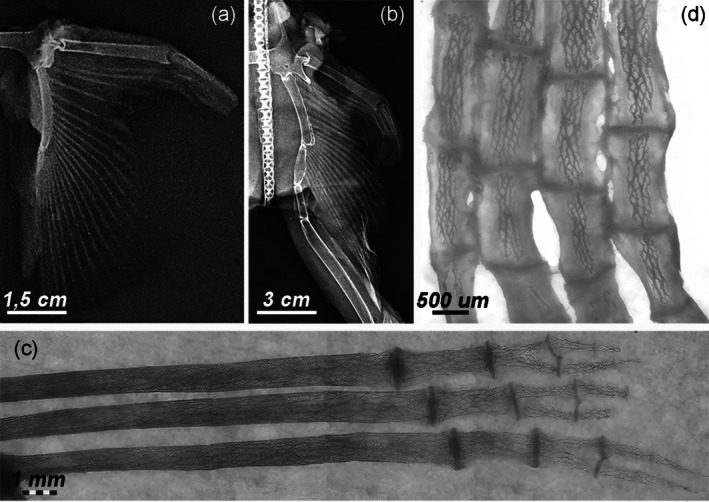
X‐rays and transillumination imaging of pelvic fins, documenting growth and the 3D‐calcified network morphology. (a, b) *Raja asterias*, specimen 4, age group B and specimen 10, age group D, showing parallel rays articulating through amphiarthroses with the pelvic pterygia. The mobile ball‐and‐socket and condylar joints exist between pelvic girdle–compound radial and the first pelvic pterygium. The first‐line radials are about 10 times the length of the distal ones, which bifurcate laterally into thin segments (not visible in X‐rays). (c) Specimen 7, age group D, transillumination of the pelvic fin, showing each radial stiffened by an internal 3D‐calcified network with disk plates thinner than those in columnar radials. Bifurcation of apical radials forming pairs of thinner segments. (d) Specimen 4, age group B, transillumination of apical, short radials showing a 3D‐calcified network similar to the first‐line radials but with a wider uncalcified cartilage muff in the younger age group.

The anterior margin of the pelvic fin was formed by a strong and thick compound radial, directly connected to the pelvic girdle via a ball‐and‐socket joint. The remaining fin skeleton consisted of 22 fin rays, connected by amphiarthroses to two basipterygia, with additional segments extending to the clasper skeleton in males. The orientation of the rays varied from anterior to posterior: (a) transverse, (b) diagonally posterolateral, and (c) almost parallel to the pterygium axis (Figure 3a,b). The number, shape, size, and arrangement of the radial rays differed markedly from those of the pectoral fin, as revealed by radiographs and transmitted light microscopy. The rays in the first line were about 10 times longer than those in the second and third lines, with each ray branching into two short series of two to three very thin apical units at the level of the fourth line (Figure 3c). The different arrangement, geometry, and pattern of mineral deposits in the two fin types (discussed in the next section, “Morphology”) made a direct morphometric comparison between the pectoral and pelvic fins of the same fish impossible (Figure 3c,d).

### 3.3 Histomorphology

**FIGURE 4:**
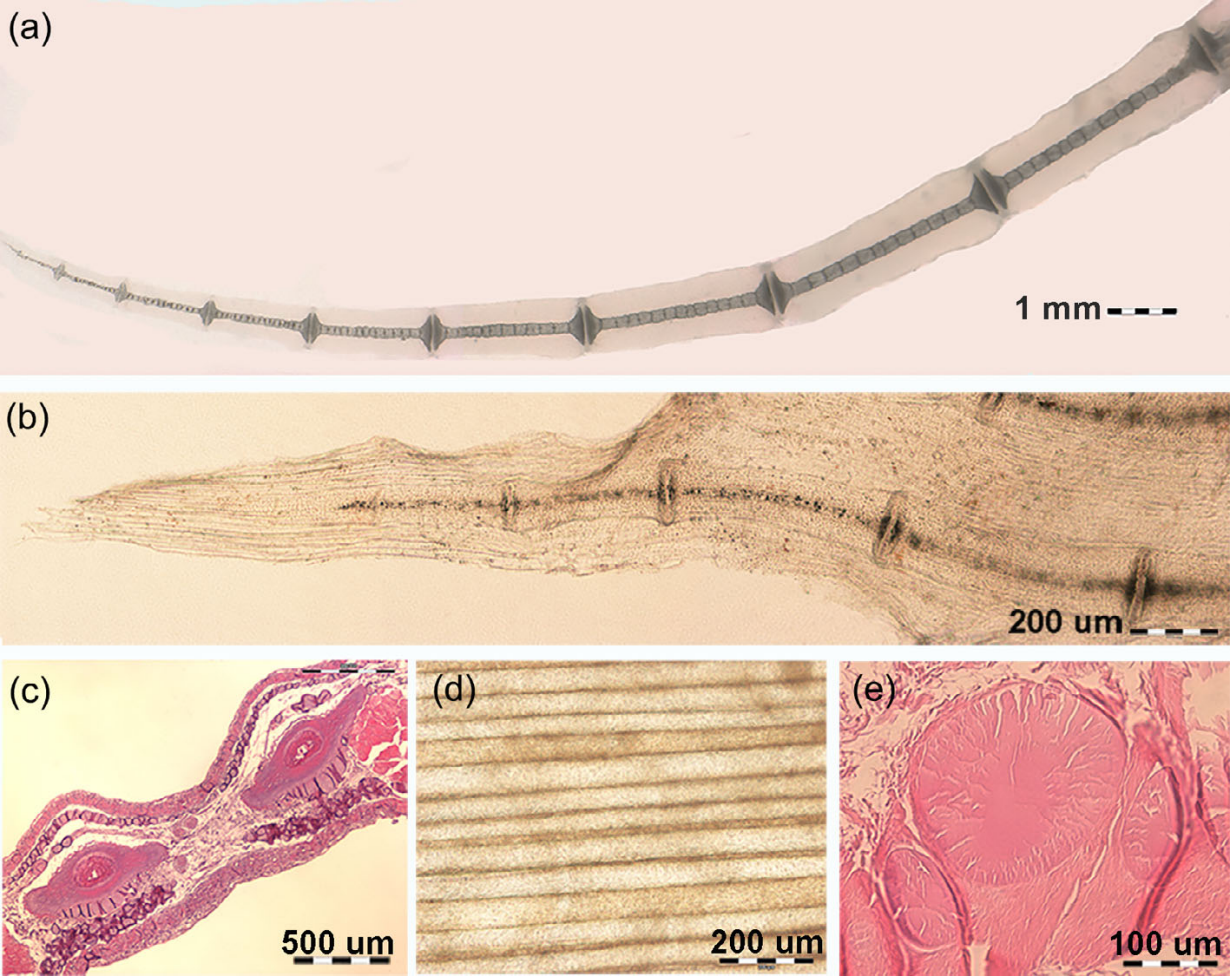
Transillumination and histomorphology of juvenile and mature *Raja asterias* pectoral fins, documenting the progression and compaction of mineral deposition (catenated pattern) and the keratin rods' structure in the fin's apical segment structure. (a) Unstained/heat‐deproteinated, 1.25×, showing the nine most lateral radials of mature *R. asterias*, with cylindrical tiles in columnar alignment. The uncalcified cartilage muff surrounds the central column. (b) Unstained/heat‐deproteinated, 10×, showing radials of juvenile *R. asterias* (specimen 3, age group B) with early mineral deposition in radials' tiles and disk plates. The fin tip reveals thin, transparent keratin rods between the dorsal and ventral skin. (c) Haematoxylin–eosin, 4×, transverse section of the apical fin displaying two parallel radials between the dorsal (up) and ventral (down) skin layers. Between these skin layers, densely packed keratin rods can be seen. (d) Unstained section in reflected light after removal of the dorsal skin layer, 10×, showing the pattern of straight and parallel rods of varying diameters (as documented in [e]). (e) Haematoxylin–eosin, 20×, transverse section showing the round shape and variable diameter of rods. The acellular keratin matrix is stained with eosin.

The cartilage anlagen of the appendicular skeleton in Batoidea differentiated from the embryonic mesenchyme (Hall, 2005), with cartilage growth soon becoming associated with mineral deposition. In the youngest *R. asterias* (e.g., age groups B and C), calcification was observed using radiography in the thicker, central segments, such as the rostrum, skull, vertebral column, girdle, and pterygia (Figure 1d). Initial mineral deposits were visible in the appendicular skeleton of the pectoral fin rays using transillumination (Figure 1a–c) and appeared as small, punctate, and dark deposits. These deposits were easier to recognize on the horizontal plane and between the plates due to the larger size and compaction of the Ca_2_PO_4_ particles (Figure 4b).

**FIGURE 5:**
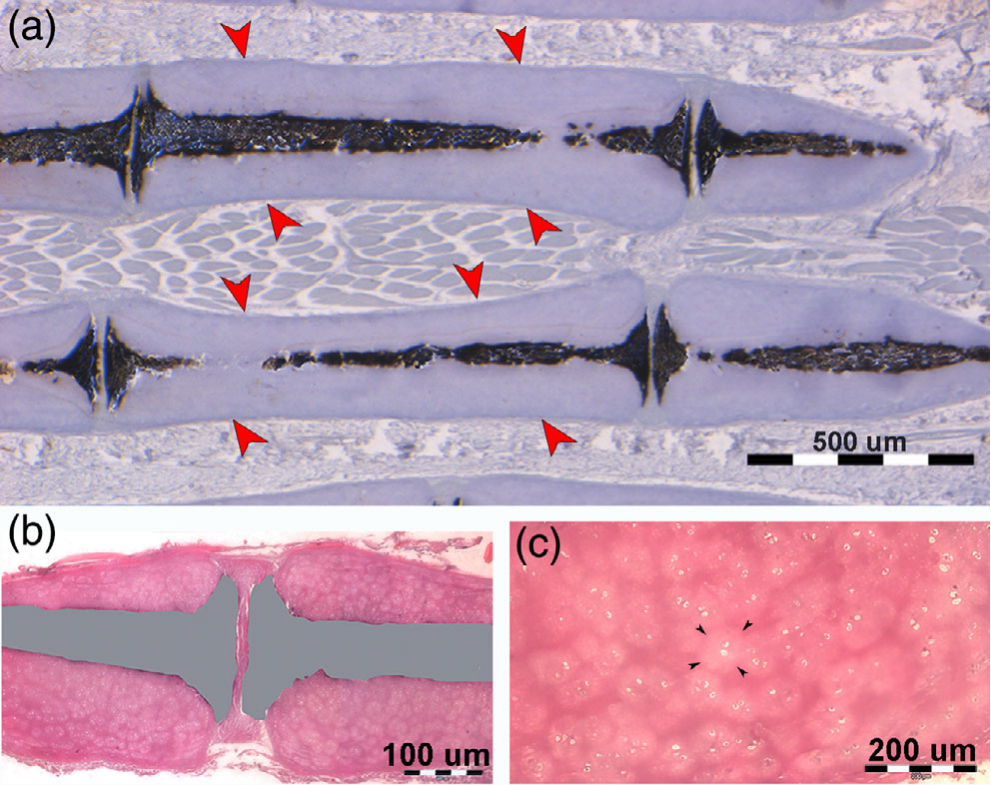
Histology of the lateral sector of the pectoral fin (undecalcified longitudinal section). (a) Resin‐embedded, undecalcified section, stained with von Kossa–Toluidine blue (4×) showing cylindrical mono‐columnar radials with a calcified central column, disk plates at the extremities, forming the interradial joints (amphiarthroses). Calcification of the columns is incomplete, with chondrocyte lacunae visible within the mineralized matrix. The uncalcified cartilage muff surrounds the calcified columns (red arrowheads), whereas gaps in the central axis are artifacts from the section plane's inclination. (b, c) Paraffin‐embedded, undecalcified section stained with haematoxylin–eosine (4×). These longitudinal sections at higher magnification illustrate the distribution of chondrocyte mitoses around the column and beneath the disk plates. (b) Shows an artificial color rendering of the torn calcified column and (c) highlights the high density of paired chondrocyte lacunae and isogenic groups within the uncalcified cartilage muff, documenting the growth pattern at the level of the radials' uncalcified cartilage cylinder (age group C).

**FIGURE 6:**
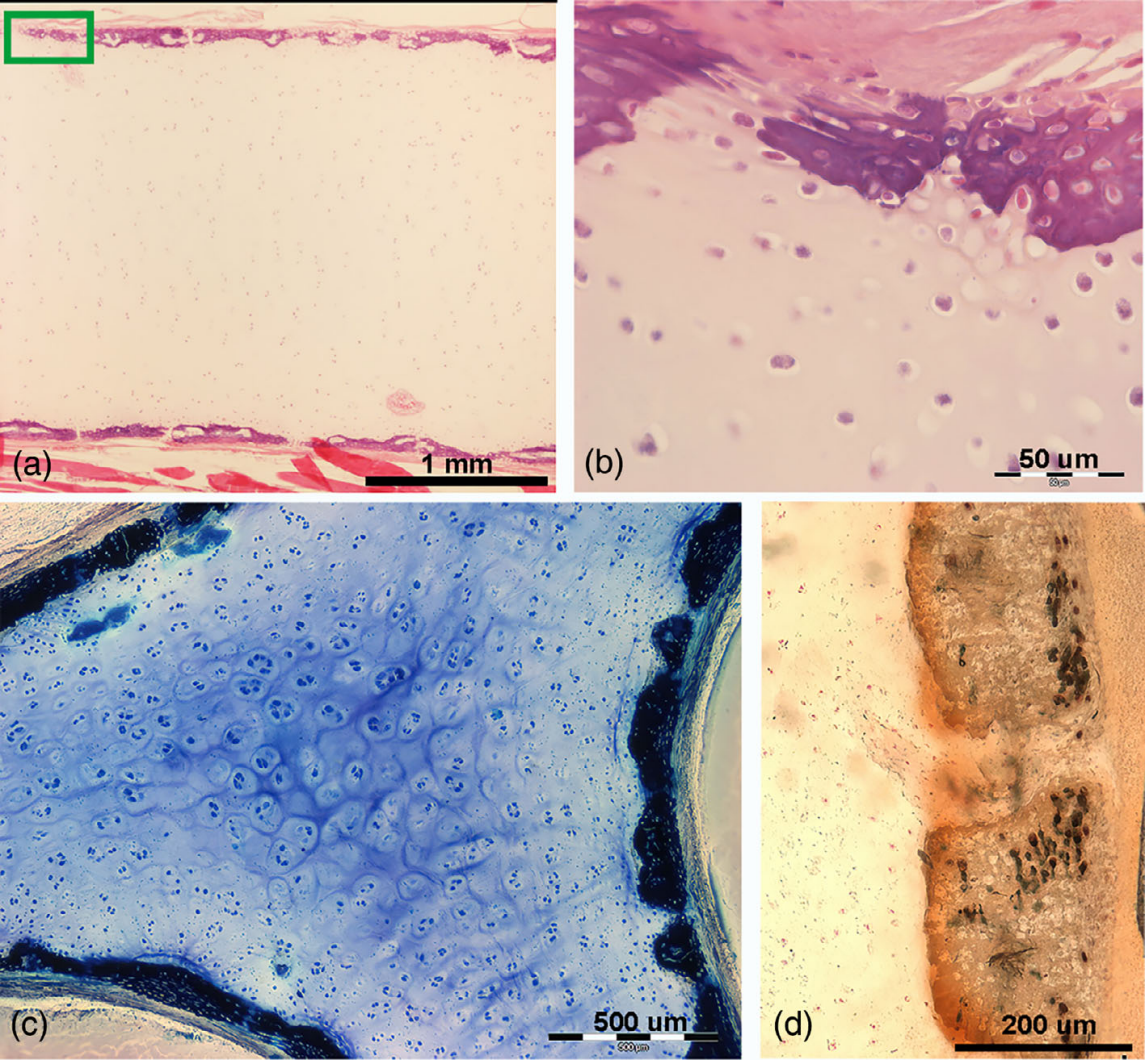
Histomorphology of larger skeletal segments (pterygium). (a) Haematoxylin–eosin, 1.25×, longitudinal section showing the early phase of “crustal” mineral deposition on the external surface of the cartilage segment. (b) Haematoxylin–eosin, 40×, transverse section highlighting the centripetal advancement of mineral deposition, with vital chondrocytes becoming embedded in the calcified matrix (green rectangle in [a]). (c) Toluidine blue, 4×, transverse section of an age group D specimen, revealing the complete “crustal” covering, with individual tesserae still evident, though some have fused over large tracts. The central core of uncalcified cartilage continues to grow, as shown by the high density of isogenic groups and aligned chondrocytes beneath the crustal layer. (d) von Kossa, 10×, transverse section showing the polygonal shape of tesserae; most chondrocyte lacunae appear empty. The dark spots are staining artifacts caused by AgNO_3_ deposits in the empty lacunae (reproduced from Pazzaglia UE et al., *Microscopy Research and Technique*, 2023; DOI 10.1002/jemt.24388, open access article distributed under the terms of Creative Commons CC, permitting unrestricted reproduction with proper citation of the original source).

In the oldest specimens of age group D, cartilage calcification was well documented in dorso‐ventral projection radiographs (Figure 1e) and in histological analysis (Figures 4a and S4). The histology of the undecalcified sections and the process of heat decalcification performed in the different age groups provided evidence of a continuous progression of mineral deposition, correlating with the growth of cartilage deposits. The individual skeletal segments do not grow by peripheral attachment of a new layer of cartilage via an outer chondrocyte layer but from within by the formation of mitotic chondroblast nests characterized by “isogenic cell groups”. These groups are scattered in the noncalcified matrix of the anlagen, and both their distribution and zonal density influence the shape of the skeletal segment into radials (Figure 5b,c), pterygia, and girdles (Figure 6c). However, mitotic activity alone was not sufficient to initiate mineral deposition around these groups, as shown in Figure 6c. Instead, the topography of calcification suggests the involvement of other factors, such as the shape and volume of the skeletal segment and the type of radials (thoracic/pelvic).

Two distinct patterns of mineral deposition were observed: (1) a “crustal pattern,” in which minerals are deposited peripherally in the longer and larger elements, such as the girdle, pterygia, and compound radials (Figures 6a and S5d); and (2) a “catenated pattern,” in which minerals are deposited centrally in the pectoral‐fin radials, forming central columns of aligned cylindrical tiles (Figures 5a and S4).

In the cartilaginous appendages, mineral deposition is centripetal in the “crustal pattern” and centrifugal in the “catenated pattern” of the pectoral‐fin skeleton (Schaefer & Summers, 2005). The calcification pattern in the rays of the pelvic fin could not be categorized into these two types, suggesting a third typology, the “3D network,” as observed in the pelvic fins (Figure 3c,d).

The structures of the “crustal” and “catenated” calcified cartilage were further emphasized by the heat‐deproteination technique, which documented the polygonal shape of the Ca_2_PO_4_ deposits in the “crustal” pattern (Figure S4d) and the cylindrical shape in the “concatenated” pattern (Figure S5a–c). Although the microscopic structure and mineral density of the calcified cartilage are similar in *R. asterias*, the calcified cartilage is softer and easier to cut with a blade compared to the calcified bone matrix (Pazzaglia, Reguzzoni, Milanese, et al., 2023).

Pectoral fins differ significantly from the pelvic fins not only in parameters such as fin area, number and orientation of rays, and length or number of radials (as shown by radiographs and morphometrics) but also in the structure of the calcified framework.

The skeletal morphology observed in the Batoidea is closely related to their swimming mechanics:

1. Pectoral‐fin radials: these structures were characterized by calcified, cylindrical tiles arranged in columns and terminating in a “plaque” at both the medial and lateral ends. On anteroposterior radiographs, the radials appeared mono‐columnar in both the medial and lateral sectors of the fin. In the medial sector, however, this mono‐columnar appearance is an artifact of the projection caused by the vertical superimposition of two parallel columns connected at their ends by a single “plaque” (Figure 2a). In the central rays, approximately in the center of the fin, the two calcified columns begin to rotate horizontally within the softer, cartilaginous cylinder of the radial. This rotation is made possible by two factors: (a) the lateral plaque (connecting the two pairs of columns) splits into two independent connections, eliminating the rigid constraint that maintains the vertical alignment of the parallel columns in the medial sector of the fin; and (b) the radial, noncalcified cartilaginous cylinder that begins to split longitudinally, forming two independent, true mono‐columnar radials (Figure S4b). In particular, the heat‐deproteination technique provided insight not only into how the calcified tiles may increase in size with growth but also into how the multi‐columnar arrangement may have increased in size with age and increased the stiffness of the skeletal segment. This increased rigidity could be due to a greater number of central columns (Figure S4) or a thicker outer calcified layer in the crustal pattern. The pectoral‐fin rays terminated in pointed calcified cartilage tips. In younger specimens, these tips showed a lower degree of calcification than in older specimens (group D). In all specimens of age groups C and D, the cartilaginous tip did not extend to the outer edge of the fin due to the adhesion of the dorsal and ventral skin. The margin consisted of thin rods (diameter of about 20–200 μm) (Figure 4b,c), which formed an extensive fan of rods or filaments between the dorsal and ventral skin layer. These rods were round in cross section, acellular, non‐birefringent under polarized light, and histochemical analysis indicated that they were composed of an ectodermal keratin‐like material (Figure 4d,e).
2. Pelvic‐fin radials: unlike the pectoral‐fin radials, the pelvic‐fin radials did not exhibit a tiling orientation with a central uni‐ or multi‐columnar pattern. Instead, their calcified cartilage framework formed a mineralized 3D lattice within the radial cartilage cylinder. The peripheral noncalcified cartilage layer, or muff, was thinner than that of the radial pectoral‐fin radials, and the thin disk plates at the extremities did not protrude beyond the mineralized lattice perimeter (Figure 3c).

## 4 DISCUSSION

Cartilage plays a pivotal role in the development of the vertebrate skeleton. It differentiates earlier than bone in the embryos of both fish and mammals, initially shaping the skeletal anlage and later guiding osteogenesis through endochondral ossification or alternative processes such as membranous ossification. The earliest evidence of embryonic tissue adapting to mechanical demands is found in the notochord, whose cells produce and deposit type II collagen, recognized as cartilaginous collagen (Hall, 2005). Although the notochord disappears early in vertebrate development, skeletal growth in most vertebrates continues via a combination of periosteal and endochondral ossification. In tetrapods, metaphyseal calcified cartilage is progressively replaced by bone until growth is complete (Pazzaglia et al., 2017; 2020).

Among jawed vertebrates (infra‐phylum Gnathostomata), the class Chondrichthyes has evolved a unique morphological strategy that diverges from the typical endochondral ossification. The anatomy and ontogeny of the mosaic‐like cartilages in chondrichthyans have been extensively studied, particularly in elasmobranchs such as the Batoidea, which exhibit distinct skeletal mineralization patterns between their bodies and fins (Schaefer & Summers, 2005; Dean et al., 2009; Seidel et al., 2016). Detailed analysis of their development, growth, and structural properties have been provided by Seidel, Blumer, Pechriggl, et al. (2017); Seidel, Blumer, Zaslansky, et al. (2017); and Seidel et al. (2021).

The functional adaptation of calcifying tissues to mechanical demands during ontogeny and organogenesis, along with the factors controlling the remodeling of calcified matrices, has spurred interest in the anatomy, histomorphology, and mechanics of the Batoidea skeleton. This model uniquely combines mineral deposition within the cartilage matrix, without subsequent remodeling, with specific locomotion mechanics within the water column (Rosenberg, 2001; Lauder & Drucker, 2004; Cerny et al., 2004; Li & Dudley, 2009; Taft, 2011; Franklin et al., 2014; Di Santo et al., 2017, 2021).

Studies using biological and material characterization techniques (Seidel et al., 2016, 2021; Seidel, Blumer, Pechriggl, et al., 2017) have uncovered the intricate structure of the mineral phase in cartilage across different elasmobranch species. These findings suggest a common damage response mechanism in the mosaic‐like structure, termed “endophytic masses (EMPS)” (Seidel, Blumer, Zaslansky, et al., 2017). However, the polygonal arrangement of “tesserae” is not the only calcified cartilage pattern in Chondrichthyes. This is further evidenced by the distinction between “crustal” and “chained” cartilage calcification patterns described by Schaefer and Summers (2005), and confirmed by comparative morphology of the pectoral, pelvic, and caudal fins in the Rajidae family (Pazzaglia, Reguzzoni, Manconi, et al., 2023).

For instance, the pectoral fins of *R. asterias* exhibit a radial pattern in the medial sector, characterized by paired tiling columns firmly connected dorso‐ventrally by unique disk plates at each end. In the lateral sector, the fin arrangement transitions into a more flexible fan of aligned single‐column radials (Pazzaglia, Reguzzoni, Manconi, et al., 2023). The pelvic fins, on the contrary, display a different architecture with fewer rays and aligned radials, where the first is significantly longer than the others, with marginal branching at the ray tip. Additionally, the internal texture of the calcified cartilage in the pectoral‐fin radials forms a 3D reticular framework, whereas only in older specimens do the longer radials begin transforming into a multi‐columnar pattern with connecting plates between the columns.

The skeletal morphology of the Rajidae family is well suited to the biomechanical demands of their environment, which can be summarized as follows:

- The central body, which includes the rostrum, skull, anterior vertebral column with synarcuals, and girdles, forms a rigid structure with minimal flexibility between its elements. These elements share a uniform “crustal pattern,” with reduced flexibility of the distal portion of the vertebral column facilitated by the intervertebral joints of the tail (Compagno, 1999).
- The symmetrical pectoral fins are articulated to the body via highly mobile ball‐and‐socket and condylar joints between the pectoral girdle and the rigid pterygial arch. The medial sector of the pectoral fins is less flexible due to the vertical overlap of paired columns, providing strong support for the percussive action of the muscles. In contrast, the lateral‐fin sector, characterized by a mono‐columnar pattern and a thin, flexible peripheral band, exclusively composed of keratin rods between the dorsal and ventral skin layers (Pazzaglia, Reguzzoni, Manconi, et al., 2023), meets the demands of undulatory dynamics.

The pelvic fins play a secondary role in forward propulsion due to their smaller surface area. Their arrangement and mineral framework differ from that of the radial pectoral fins and larger body segments with the “crustal” pattern.

To the best of our knowledge, this study is the first to report the internal mineral framework of the pelvic fins, referred to as the “3D network,” within the skeletal structure of Batoidea. The cartilage forms smaller, finer calcified aggregates than those found in the “radial tiles” or “tesserae,” which fill most of the radial cylinder, leaving only a thin peripheral layer of noncalcified cartilage in mature specimens. This morphology suggests that the majority of the pliability of these fins is due to the medial connection between the pterygium and the first long radialis, rather than the connection between the shorter radialis. The morphology, geometry, and low muscle mass of the pelvic fins indicate that they contribute minimally to forward thrust generation, instead performing a passive glide when the fish sinks in the water column or turns laterally (Rosenblum et al., 2011).

Rajiform swimmers move by undulating the distal parts of their pectoral fins, with several waveforms visible on the fin at the same time. The kinematics of Rajidae pectoral fins, characterized by a combination of “fluttering” and “undulating” movements, have been analysed in vivo by Rosenberg (2001) and Blevins and Lauder (2012). Di Santo et al. (2017) documented the effects of speed on pectoral‐fin deformation and the convergence of undulatory swimming kinematics across diverse Batoidea species in the small skate *Leucoraya erinacea* (Di Santo et al., 2021). Taft (2011) and Franklin et al. (2014) further explored the relationship between phylogenetic variation and geometric morphometrics. The mechanics and variation in fin geometry suggest a crucial role for fin column patterns, highlighting the unresolved question of how growth and mineral deposition are regulated in these calcifying matrices.

In conclusion, this study offers the first detailed description of the internal mineral framework of the pelvic fins, known as the “3D network,” in *R. asterias*. This species belongs to Batoidea, a subdivision of the class Chondrichthyes, which encompasses cartilaginous fishes such as sharks, rays, and skates. The unique morphology, characterized by smaller, finer calcified aggregates compared to the “radial tiles” or “tesserae,” indicates that most of the pliability in the fins is derived from the medial connection between the pterygium and the first long radialis. This suggests a structural adaptation that prioritizes flexibility rather than rigidity. Additionally, the geometry and low muscle mass of the pelvic fins imply that they play a minimal role in forward thrust generation. Instead, these fins function more passively, contributing to gliding movements when the fish sinks or turns laterally in the water column.

## AUTHOR CONTRIBUTIONS

Conceptualization: Ugo E. Pazzaglia and Marcella Reguzzoni. Methodology: Piero A. Zecca. Software: Piero A. Zecca. Validation: Fabrizio Serena and Cecilia Mancusi. Formal analysis: Guido Zarattini. Investigation: Fabrizio Serena. Resources: Ugo E. Pazzaglia. Data curation: Marcella Reguzzoni. Writing—original draft preparation: Ugo E. Pazzaglia and Genciana Terova. Writing—review and editing: Ugo E. Pazzaglia, Genciana Terova, and Fabrizio Serena. Visualization: Genciana Terova. Supervision: Ugo E. Pazzaglia. Project administration: Genciana Terova. Funding acquisition: Genciana Terova. All authors have read and agreed to the published version of the manuscript.

## FUNDING INFORMATION

This research was funded by the “Fondo Comune di Ateneo per la Ricerca—University of Insubria, Italy (FAR_2024 to Genciana Terova).

## CONFLICT OF INTEREST STATEMENT

The authors declare that they have no competing financial interests or personal relationship that could have influenced the work reported in this paper.

## Supporting information


**Supplementary Figure S1.** Mechanical models of pectoral and pelvic fins, illustrating the larger surface area of the pectoral fins. Although both fins share similar mobile joints (diarthroses) with their respective girdles, only the pectoral fins enable wide flapping movements, developing different kinematics due to differences in surface area, muscle mass, flexibility, radial number, length, shape, and the inner calcified structure, which collectively drive the flapping‐undulatory movement. The only mobile ball‐and‐socket joint in the pectoral fin is between the pelvic girdle–compound radial, whereas fin rays form amphiarthroses with pelvic pterygia.

**Supplementary Figure S2.** Graphical representation of radial length regression in the mono‐columnar rays (a) from the oldest *Raja asterias* specimen (*n* = 10), alongside the corresponding number of tiles per radial (b) in the five central rays. The mono‐columnar radials do not result from bifurcation in the medial‐fin sector but from the horizontal plane rotation of the two paired columns at the transition between the medial and lateral sectors. The observed discrepancy between radial length regression and the number of calcified tiles indicates that these two parameters are not simply linearly correlated (modified from Pazzaglia et al., 2023, doi: 10.1111/joa1.881).

**Supplementary Figure S3.** Interradial joint histology (resin‐embedded, undecalcified longitudinal section, stained with methylene blue, 20×).
The fibrous space between the disk plates of the interradial joint is marked (#). The branches of the columns that support the disk plate and the solid, basal tesserae (*) exhibit a morphology similar to the crustal covering found in larger skeletal segments, such as pterygia and girdles. Chondrocytes are arranged in rows (red arrows) within the uncalcified cartilage between the branches and near the calcified cartilage.

**Supplementary Figure S4.** Unstained, heat‐deproteinated (400°C) longitudinal thick sections of the pectoral fin's lateral sector and transition zone, post‐dissection of skin and muscles. (a) (Transillumination, 4×): transition from multi‐columnar radials in the medial sector to mono‐columnar radials in the lateral sector, composed of aligned, calcified tiles. Red arrowheads indicate the edges of the uncalcified cartilage muff. (b) (Transillumination, 4×): mono‐columnar radials in the lateral sector. (c) (Transillumination, 10×): early mineral deposition forming aligned tiles, with some tiles undergoing fusion. Enlarged tile details are shown at 20× in the upper left corner. Chondrocyte lacunae are visible within the uncalcified cartilage muff. Red arrowheads delineate the borders of the uncalcified cartilage, with mineral deposition predominantly following a longitudinal orientation (catenated pattern) rather than eccentric calcification. (d) Phase‐contrast image corresponding to (c) highlighting the chondrocyte lacunae within the uncalcified cartilage.

**Supplementary Figure S5.** Heat‐deproteinated (1200°C) radials and pterygia under reflected light microscopy. The images demonstrate the transformation of Ca_2_PO_4_ into hydroxyl apatite within the tesserae and tiles. (a) Compact and flat surface of the disk plate, observed from the inner side of the interradial joint. (b) Opposite view showing the cutoff branches of the columns that support the disk plate. (c) Lateral view of the disk plate with the branching tile columns extending to support the plate at the radial extremities. (d) External view of the polygonal tesserae of the pterygia and the connecting amphiarthrosis.

## Data Availability

The data that support the findings of this study are available from the corresponding author upon reasonable request.
