# Supplementary figures and images for: Growth and mechanical correlations of calcified cartilage in Batoidea: A histomorphological study using the Raja asterias model
Source: J Fish Biol. 2025 Jan 2;107(1):23–33. doi: 10.1111/jfb.16037 (PMC12327180; doi:10.1111/jfb.16037)

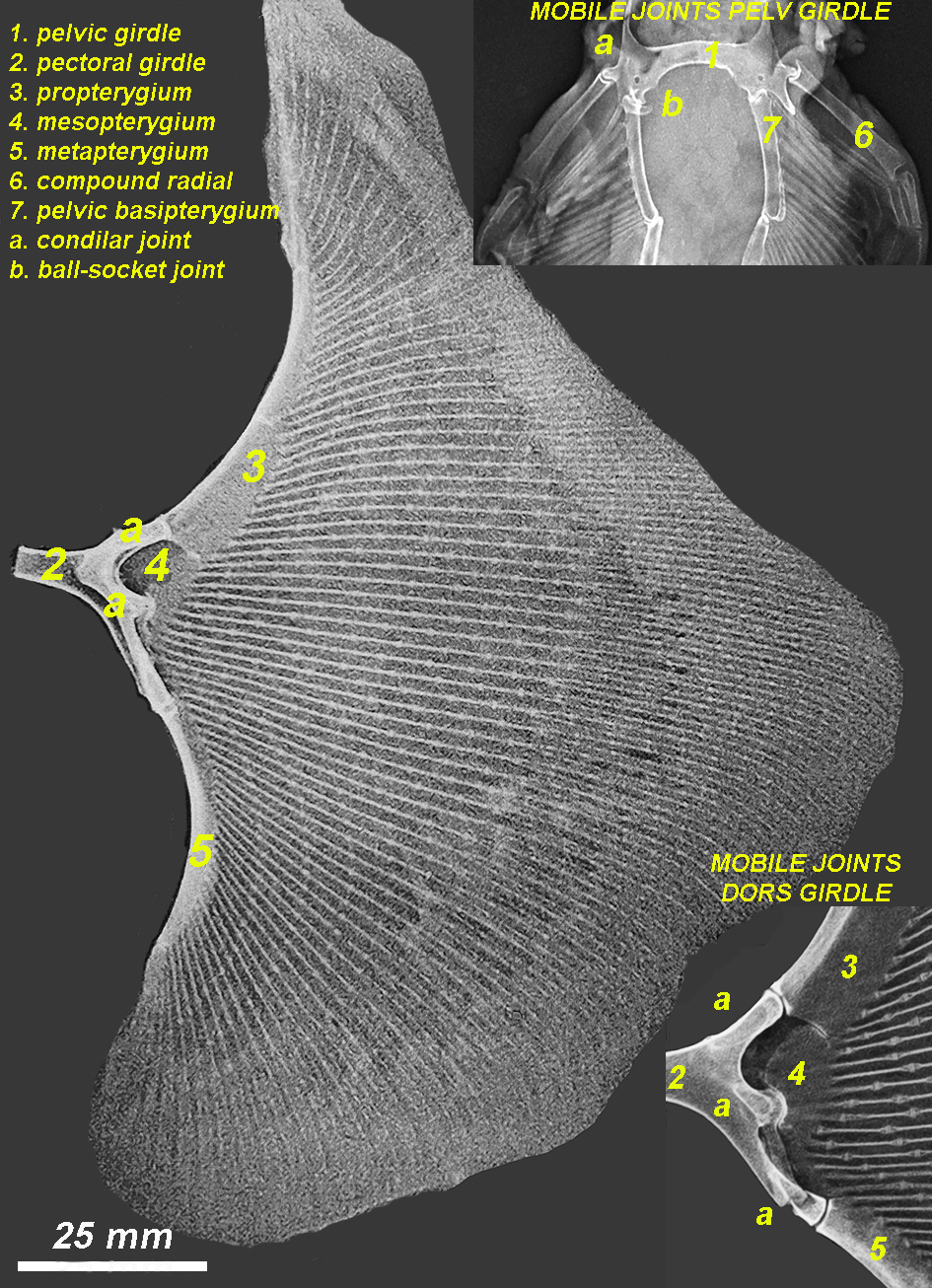

Supplement: Supplementary file 1 — Supplementary Figure S1. Mechanical models of pectoral and pelvic fins, illustrating the larger surface area of the pectoral fins. Although both fins share similar mobile joints (diarthroses) with their respective girdles, only the pectoral fins enable wide flapping movements, developing different kinematics due to differences in surface area, muscle mass, flexibility, radial number, length, shape, and the inner calcified structure, which collectively drive the flapping‐undulatory movement. The only mobile ball‐and‐socket joint in the pectoral fin is between the pelvic girdle–compound radial, whereas fin rays form amphiarthroses with pelvic pterygia. [file JFB-107-23-s002.tif]

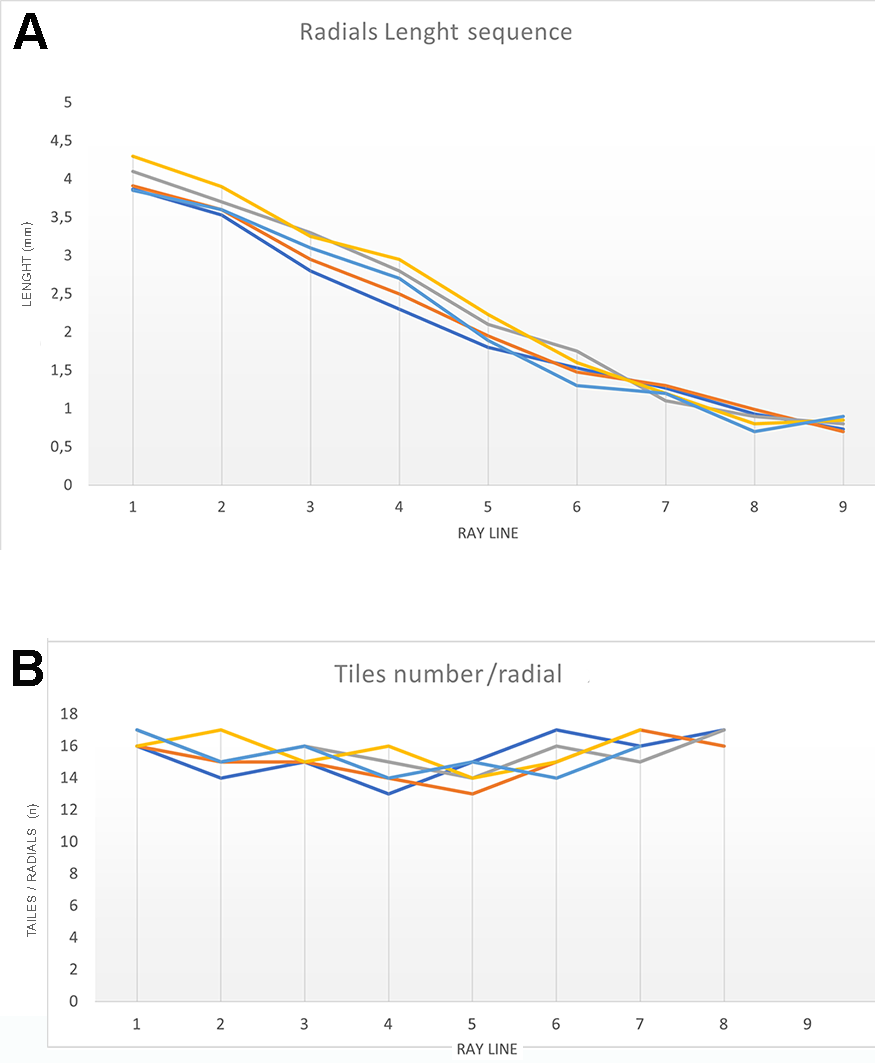

Supplement: Supplementary file 2 — Supplementary Figure S2. Graphical representation of radial length regression in the mono‐columnar rays (a) from the oldest Raja asterias specimen (n = 10), alongside the corresponding number of tiles per radial (b) in the five central rays. The mono‐columnar radials do not result from bifurcation in the medial‐fin sector but from the horizontal plane rotation of the two paired columns at the transition between the medial and lateral sectors. The observed discrepancy between radial length regression and the number of calcified tiles indicates that these two parameters are not simply linearly correlated (modified from Pazzaglia et al., 2023, doi: 10.1111/joa1.881). [file JFB-107-23-s001.tif]

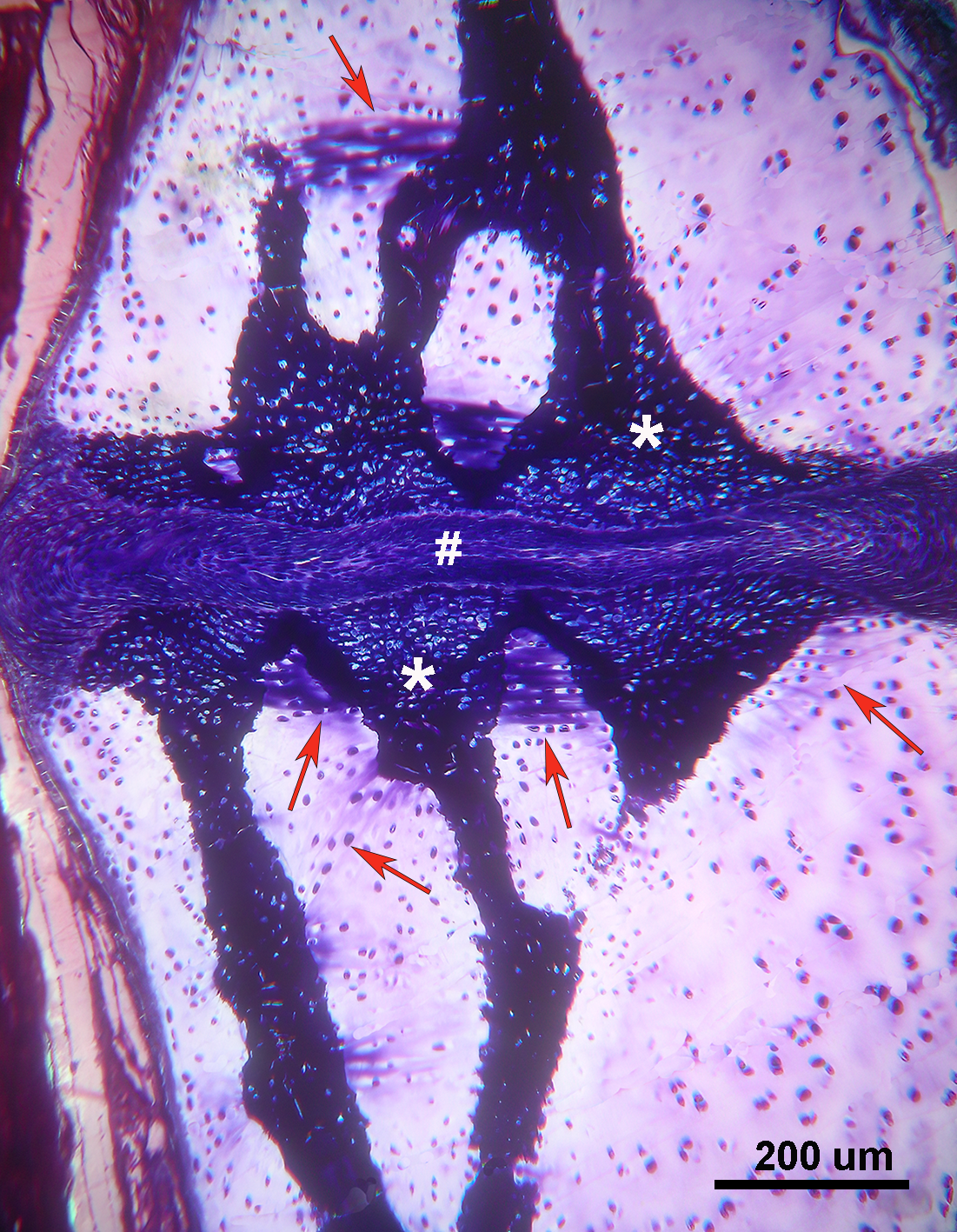

Supplement: Supplementary file 3 — Supplementary Figure S3. Interradial joint histology (resin‐embedded, undecalcified longitudinal section, stained with methylene blue, 20×). The fibrous space between the disk plates of the interradial joint is marked (#). The branches of the columns that support the disk plate and the solid, basal tesserae (*) exhibit a morphology similar to the crustal covering found in larger skeletal segments, such as pterygia and girdles. Chondrocytes are arranged in rows (red arrows) within the uncalcified cartilage between the branches and near the calcified cartilage. [file JFB-107-23-s004.tif]

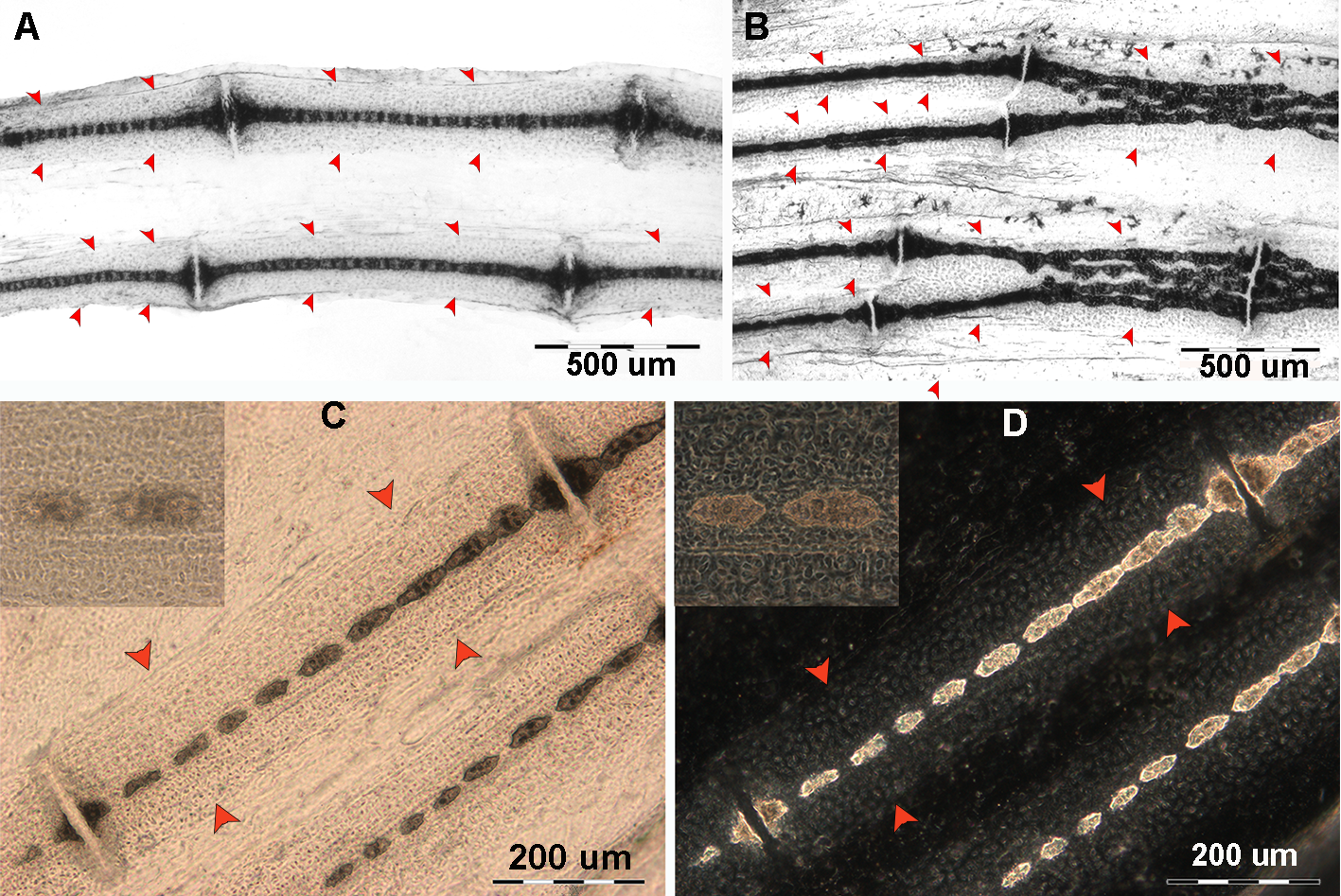

Supplement: Supplementary file 4 — Supplementary Figure S4. Unstained, heat‐deproteinated (400°C) longitudinal thick sections of the pectoral fin's lateral sector and transition zone, post‐dissection of skin and muscles. (a) (Transillumination, 4×): transition from multi‐columnar radials in the medial sector to mono‐columnar radials in the lateral sector, composed of aligned, calcified tiles. Red arrowheads indicate the edges of the uncalcified cartilage muff. (b) (Transillumination, 4×): mono‐columnar radials in the lateral sector. (c) (Transillumination, 10×): early mineral deposition forming aligned tiles, with some tiles undergoing fusion. Enlarged tile details are shown at 20× in the upper left corner. Chondrocyte lacunae are visible within the uncalcified cartilage muff. Red arrowheads delineate the borders of the uncalcified cartilage, with mineral deposition predominantly following a longitudinal orientation (catenated pattern) rather than eccentric calcification. (d) Phase‐contrast image corresponding to (c) highlighting the chondrocyte lacunae within the uncalcified cartilage. [file JFB-107-23-s005.tif]

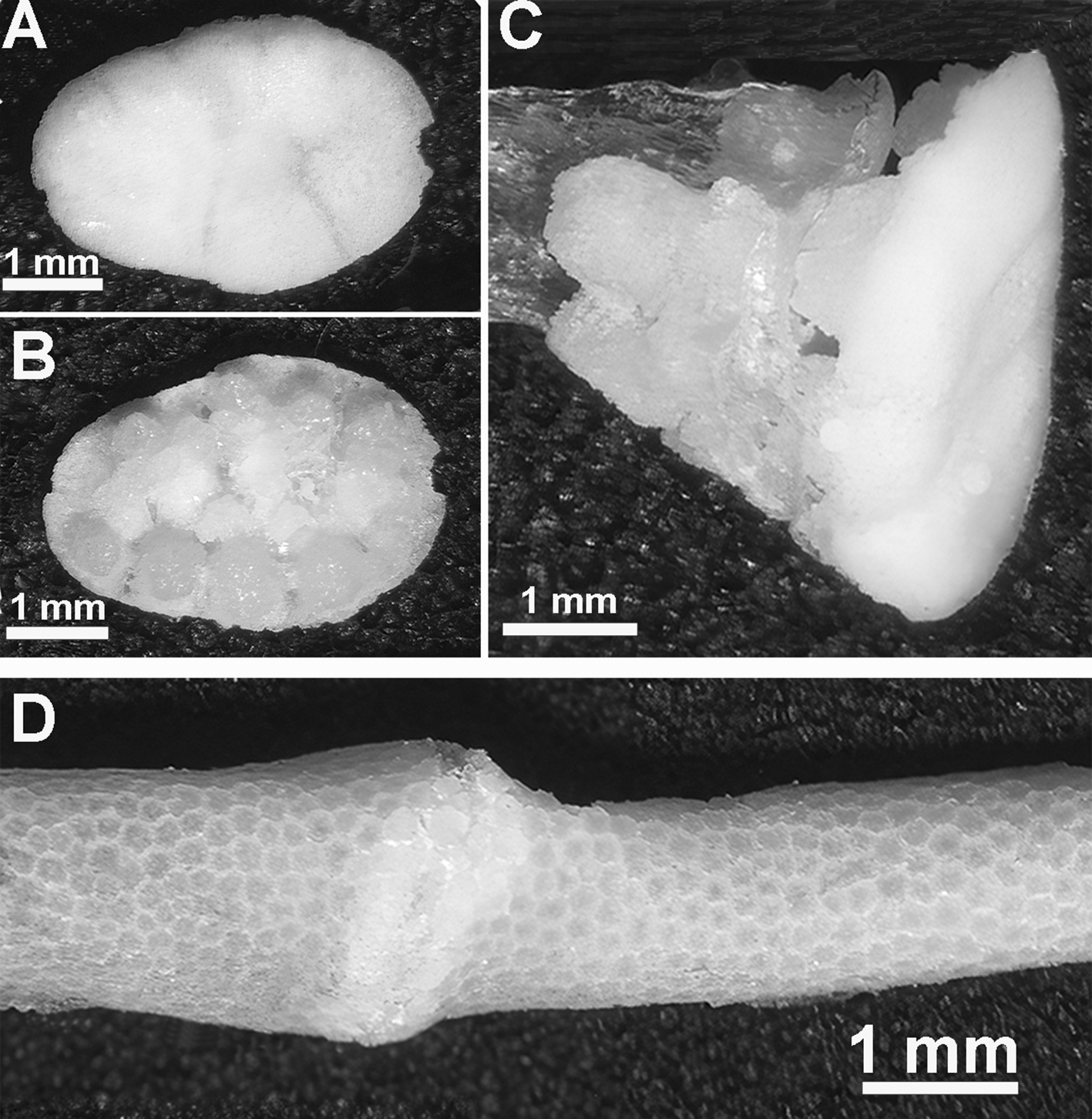

Supplement: Supplementary file 5 — Supplementary Figure S5. Heat‐deproteinated (1200°C) radials and pterygia under reflected light microscopy. The images demonstrate the transformation of Ca2PO4 into hydroxyl apatite within the tesserae and tiles. (a) Compact and flat surface of the disk plate, observed from the inner side of the interradial joint. (b) Opposite view showing the cutoff branches of the columns that support the disk plate. (c) Lateral view of the disk plate with the branching tile columns extending to support the plate at the radial extremities. (d) External view of the polygonal tesserae of the pterygia and the connecting amphiarthrosis. [file JFB-107-23-s003.tif]
